# Experimental, therapeutic and natural transmission of *Plasmodium vivax* tertian malaria: scientific and anecdotal data on the history of Dutch malaria studies

**DOI:** 10.1186/1756-3305-6-19

**Published:** 2013-01-18

**Authors:** Jan Peter Verhave

**Affiliations:** 1 Department of Medical Microbiology, Radboud University Medical Centre, Nijmegen, 6500 HB, The Netherlands

**Keywords:** *Plasmodium vivax*, Experimental infection, Neurosyphilis, Long latency, Relapse

## Abstract

When *Plasmodium vivax* tertian malaria was prevalent in The Netherlands, the use of therapeutic malaria for the treatment of neurosyphilis patients presented an opportunity for biological studies of the parasite’s behaviour, in healthy volunteers. One unexplained phenomenon was the long latency between natural exposure to a single infected mosquito and the appearance of clinical signs (average 8 months). Dutch studies with volunteers and syphilis patients, suggested that hundreds of sporozoites transmitted by several mosquito bites were enough to provoke an early attack, known from tropical vivax-malaria. Sporozoites appeared to be programmed either to delay their pre-erythrocytic development or to proceed to an early attack within three weeks. The number of infectious bites also determined the relapse rate and the number of relapses after a primary attack. Analyses of primary cases and relapses from the previous year were used to predict the incidence for the next year. These historic findings fit well with recent studies on genotyping of blood stages during primary attacks and relapses. External factors (i.e. the milieu inside the human host) may trigger hypnozoites to reactivate, but predetermined periods of latency should also be considered.

## Review

The biology of *Plasmodium vivax* is very complex. Various existing strains appear to have different behaviour patterns in patients. Some cause primary attacks within weeks, others remain latent for months. Relapse patterns also differ.

Until recently, these differences could only be studied in volunteers or patients suffering from general paralysis of the insane (GPI, neurosyphilis, caused by the spirochete *Treponema pallidum*) who were deliberately exposed to infected mosquitoes. The extended bouts of fever of this so-called “malaria therapy” killed the spirochetes and improved the patient’s condition. During the second decade of the twentieth century, the effectiveness of malaria-induced fevers triggered the set-up of elaborate systems for infecting and maintaining anopheline mosquitoes in several European countries and in the USA. The constant need for infected mosquitoes or parasitized blood required a rigid organisation in the mental institutions where these patients were being treated. An additional advantage of the therapeutic infections was the detailed study of the transmission biology of *P. vivax*.

In this article the studies of experimental and natural malaria that were carried out in Amsterdam and its surroundings, are analysed. The aim is to show how these studies arose and how the scientists involved put forward far-reaching suggestions that appear to be surprisingly compatible with modern research.

*Plasmodium vivax* tertian malaria was endemic in The Netherlands until the middle of the twentieth century. The Dutch strain was the last remnant of a once common *P. vivax* covering Northern Europe and Asia, adapted with its long latency to relatively cold winters. It became extinct in Europe in 1960 (though The Netherlands was declared malaria-free only in 1970).

Malaria was endemic, particularly in the province of North-Holland, north of Amsterdam [1]. The malaria research group had its home in the city, and consequently, a centre for therapeutic malaria (or malaria therapy) was installed in a nearby neurological clinic, as carriers of parasites were readily available in the area.

Pieter C. Korteweg described the initial remittent fevers in vivax-malaria, that since then bear his name [2]. Patients without previous exposure to malaria, developed quotidian fevers with extremely low parasite densities, and treatment with quinine took several days to resolve the symptoms. Only after some 2–6 days without treatment did parasites and fevers change into the familiar intermittent pattern (48 hours). Patients with previous experience developed intermittent fevers without the Korteweg fevers, and with easily detectable parasitaemias, both of which quickly disappeared after treatment [3].

Another important finding was that sporozoite-induced infections could give rise to relapses, which never occurred after inoculation of infected blood.

The peak of naturally infected patients in the Dutch summer did not coincide with the period when mosquitoes were found to be infected. The population was still building- up outdoors, and few mosquitoes acquired an infection. In a search to find the crucial period for survival of *P. vivax* in a temperate climate, it turned out that the peak of infected mosquitoes occurred in the autumn when female mosquitoes were seeking shelter inside houses, and where they kept taking blood meals. When healthy parasite carriers were present (mostly children with sufficient gametocytes in their blood) the mosquitoes became infected, and eventually infectious [4,5]. The majority of people infected during autumn experienced their first attack of vivax-malaria around the following May, without any earlier sign of infection. In 1902 Korteweg had already formulated a hypothesis [6,7], which he further elaborated with reference to the long latency, when malaria therapy of GPI-patients was about to begin (and of which he was in charge after his retirement as a general practitioner) [8]. The hypothesis was that infectious bites in autumn led to a first clinical attack during the following spring or summer, but it required further proof. Swellengrebel and Schüffner, respectively Professors of Parasitology and Tropical Medicine at the Institute of Tropical Hygiene in Amsterdam, decided in 1928 to perform experimental infections on healthy volunteers that would imitate natural conditions (i.e. the use of one infectious mosquito, and not many as used for GPI-patients) [5]. The results of this experiment and its follow-up provide complementary sources of information and consideration of a recent publication by Nicolas White, a comprehensive study on latency and relapses of *P. vivax* in which he cited some of the work of Korteweg and Swellengrebel, which he considered as brilliant [9].

### *P. vivax* and long latency

To test the long latency hypothesis, healthy volunteers were exposed to infected mosquitoes. *Anopheles atroparvus* mosquitoes were infected from a GPI-patient carrying many gametocytes, and the mosquitoes developed 10–50 oocysts. When the researchers predicted that sporozoites would be present in the salivary glands, Schüffner and Swellengrebel, Mrs Swellengrebel, Korteweg and some other colleagues and collaborators volunteered to be bitten by one or two mosquitoes that subsequently appeared to be positive with sporozoites. After their exposure in September-November 1928, none of them developed a parasitaemia or a clinical attack within weeks, but all of them came down with fever and parasites in their blood, some 8 months later, in the next year.

Because the Health Division of the Rockefeller Foundation supported the experiment, Swellengrebel informed its representative in Europe, Dr George K. Strode on July 31^st^, 1929: “We all had rather sharp attacks as we wished to be sure that they were not only parasites but also a good fever. I was just a little uneasy about my wife who is going to be confined in October.” (*, an attack might affect pregnancy and provoke a relapse, see below). Schüffner and Swellengrebel documented the results of 6 volunteers for publication [10-12]. The hypothesis was now a proven fact and in complete agreement with what had been observed in natural infections. Satisfied, the two leaders departed for a long trip to British India on behalf of the Malaria Commission of the League of Nations [5].

During this travel, Schüffner received a letter from another participant in the infection experiment. This volunteer (medical doctor C. Delprat) had exposed himself to mosquitoes several times in November 1928. He became feverish on 26th August, 1929, and parasites appeared 4 days later, after which he started taking quinine. On 3rd September, whilst driving a car, he experienced a severe pain in his left side, passed out and was admitted to hospital. He reported the diagnosis (rupture of the spleen with some internal bleeding, due to vivax-malaria) to Schüffner in Bombay, with the strong request to keep it secret, because he had never informed his wife about his voluntary participation in the experiment… Swellengrebel felt relieved that the experiment had not resulted in the death of this volunteer (**).

Interestingly, in the published reports, written-up after six people had developed malaria, the authors mentioned all participants by name and their previous malaria history. However, in his book *Malaria in The Netherlands*[13], Swellengrebel recounted the experiment and mentioned in a footnote an eighth volunteer whose case was not published before, because of personal reasons (page 150). This must have been Dr. Delprat. The data of a seventh volunteer also did not appear in the publications and the details remain unknown. In the 8 volunteers, the average latent period was 8 months and 15 days, which coincided with *P. vivax* patients infected with the strain that was indigenous in England*,* whose latent period amounted to 8 months and 27 days.

Six GPI-patients exposed to 8–13 bites of mosquitoes from the same batch developed fever and parasitaemias within three weeks, but such results were not always so coherent. Taken together with the fact that the Dutch *P. vivax* often took too long before the first attack the therapeutic treatment was not a smooth procedure. Moreover, the GPI-patients did not experience enough of the necessary chills if they had had malaria before. Apart from a few trials with *P. malariae*[14], the routine exposure of paralytic patients was continued with mosquitoes infected with the Madagascar strain of *P. vivax* (supplied by Colonel S.P. James, who directed a similar malaria-therapy program at The Horton Hospital, Epsom UK). This M-strain almost invariably resulted in short incubation periods of about twelve days, rather than the three weeks of the indigenous Holland (H) strain and also caused more fever. In addition, mosquito infections with the M-strain were more successful (60%) than with the H-strain (40%), even when patients had experienced a natural or therapeutic infection with the same strain before. The M-strain provided a welcome solution to the poor rates of therapeutic efficacy of the H-strain, even when many mosquitoes were allowed to bite. Korteweg reported many details about these two strains, used for therapeutic purpose [15], and demonstrated that the H-strain did not protect against the M-strain [16].

A second infection experiment began in September 1931 with 15 healthy volunteers [17] exposed to 5–12 mosquitoes infected with the M-strain. After three weeks all but one volunteer came down with primary attacks in September or October; the one case of protracted incubation appeared in April 1932. This was consistent with the finding of Korteweg, that 93% of the GPI-patients infected with the M-strain got malaria after a short incubation. The lesson learned was that the M-strain led to short incubations, at least after 5–12 infected mosquitoes, and was thus far more suitable for GPI-patients.

The results of an experiment of therapeutic malaria, using 3–5 mosquitoes, infected with the H-strain was intermediate: half of the GPI-patients developed symptoms and blood parasites after a short incubation. Swellengrebel concluded that after several bites, and thus the inoculation of more sporozoites, the first attack tended to appear after a few weeks. This was convincingly demonstrated by Korteweg and later by Winckel [18], who succeeded him as malaria consultant for the malaria therapy program.

However, a straight relationship between numbers of exposures to the bites of infected mosquitoes and short incubation of the H-strain in 79 GPI-patients could not be demonstrated [13], at least not per experiment (Table 1). Even heavy infections often led to a long latency, much to the disappointment of Korteweg. These infections were performed throughout the year. 

**Table 1:** Relationship between incubation period and number of biting mosquitoes infected with the H-strain

**No. mosquitoes used**	**No. patients exposed**	**No. with short incubation**	**No. with long incubation**
3-5	14	7	7
6-10	17	13	4
11-20	18	13	5
21-30	15	7	8
> 30	15	9	6
	79	49 (62%)	30 (38%)

Abraham de Buck later confirmed and extended the observations of Korteweg: 42% of another 85 therapeutic infections, with exposure to at least 25 infected mosquitoes per neurosyphilitic person, gave no immediate parasitaemia and he attributed this to protracted incubation [19]. In addition, De Buck (who had been one of the volunteers in the first experiment) concluded that such protracted periods are not caused by seasonal influence, but by some inherent quality of the H-strain and infections with few mosquitoes (no longer practised at the time) would have resulted in similar protracted incubations, regardless of the season.

Finally, Winckel summarised the results of 87 GPI-patients receiving many bites from mosquitoes infected with the H-strain: 59% suffered a clinical attack within three weeks and the rest had a delayed attack [18].

Thus, three sets of GPI-patients, exposed throughout the seasons to many mosquitoes, infected with the H-strain gave similar results: little over half of the patients developed an early infection. No direct relationship with the number of bites (sporozoite inoculum) was found. In fact, it is plausible that if more volunteers had been used in the initial experiment with one mosquito, some may have experienced an early infection, a possibility not considered in the publications.

The conclusion of the Dutch malariologists, that the size of the sporozoite inoculum of the H-strain determined the chance of early attacks, is not easily reconcilable with the idea of sporozoites genetically predisposed for long or short latency, and of the oocysts they originated from. But at least one may assume from the experiment with one mosquito that the number of short latency oocysts was in the minority (in contrast to those of the M-strain). When an early attack occurred, at least one oocyst and its successive sporozoites must have been predetermined for short latency. With larger inocula, the clinical outcome shifted and the chance of one or a few “short” oocysts releasing their sporozoites to the salivary glands, caused more short latency attacks.

An example with numbers: suppose a mosquito with 50 oocysts, each producing 4000 sporozoites and 25% ending up in the salivary glands [20]; if one oocyst has a “short” make-up, 1000 of the 50,000 sporozoites are “short”. If 100 sporozoites are transmitted per bite, two of them are “short”. With one mosquito bite the likelihood may be low that these two cause an early attack, but with more infective bites per person, the chance increases. Whatever the breaking point, one may assume a mix of genetically different zygotes/oocysts.

GPI-patients may later also have suffered an additional long latency attack, but information is obscured by the immune response mounted during extended periods of untreated fever.

### Relapses in *P. vivax*

The phenomenon of a reappearing parasitaemia with clinical signs after a previous attack and a healthy period without parasitaemia in-between, is called a relapse. At the time these experiments were carried out, researchers only had a vague idea that parasites might have some resting phase outside the blood and in some organ (which we know now to be the liver), where they are unaffected by quinine. The reactivation signal was, and still is, an enigma [21]. The question arises whether a relapse after a first attack (in the M-strain and others) and a first attack, long after exposure to an infected mosquito (more common in the H-strain), are the same phenomenon.

Unfortunately, the Dutch researchers did not report about the relapses of the eight volunteers with their latent periods of over 8 months (nor of the GPI-patients infected with the H-strain). However, Swellengrebel, in his letters to his wife from British India reported about his own relapses (**). The two travelers were prepared for some follow-up because of the experimental origin of the infection. Indeed, Schüffner suffered two relapses, one before embarking for India and one in Bombay. Swellengrebel described the relapses he experienced himself and reminded his pregnant wife to note carefully the details of her relapses; apparently she was spared. After the experimental exposure to a mosquito on October 30^th^ and December 5^th^, 1928: 

• The primary attack with fever and 2 parasites/1000 leucocytes started on July 20^th^ and lasted as a quotidian intermittent fever with up to 124 parasites, until treatment with quinine terminated this episode on the 26^th^.

• The first relapse started on August 9^th^, with 72 parasites per 1000 leucocytes the following day and a sharp fever and sweating, despite taking quinine starting 24 hours earlier. On August 12^th^ the fever subsided to 36.0°C although he felt still a bit shaky.

• His second relapse began exactly 3 weeks after the first one. On August 30^th^ his temperature rose to 38.5°C with a parasitaemia of 36 per 1000 leucocytes. He took quinine and the fever disappeared the following day (quotidian had become tertian).

• He expected the third relapse to occur on the 21^st^ September, but it came on the 25^th^. He fainted and had a classical attack with chills, heat and sweating; he felt weak and had a palpable spleen, which stayed enlarged until the end of the month.

Assuming that the quinine always cleared all parasites from the blood, this case history shows that, even after long latency, more than one relapse could occur. Other similar observations on natural Dutch malaria exist, but this case is special, because of the professional observations of the phenomena on himself. Swellengrebel was apparently accustomed to the constant periodicity of relapses that also could occur after a short latency primary attack [9]. A few of the scarce details on relapse patterns in Swellengrebel’s publications are as follows.

The relapse rate of the experiment with the 15 volunteers, exposed to 5–12 mosquitoes infected with the M-strain, was almost 100%, compared with 50% after one bite. There were 39 relapses from December to next November the following year, with a peak in May. The authors wondered whether the one volunteer out of 15 with a protracted attack might have had an unobserved early infection that made it a true relapse. But no, all relapses manifested themselves with intermittent fever, whilst the one long latency attack came with an initial remittent Korteweg fever, which excluded an earlier parasitaemia [17].

**Figure 1:**
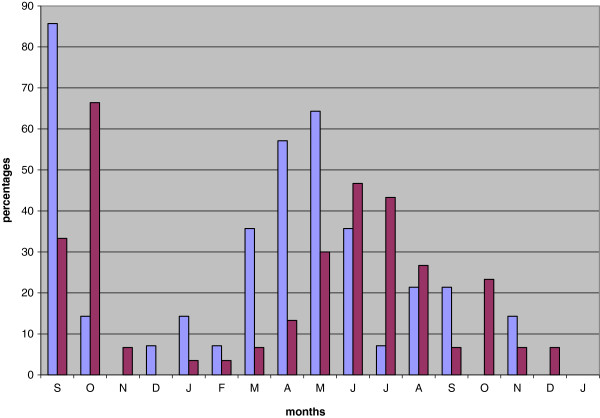
**Comparison of experimental infections (blue n=15, M-strain) and natural primary autumn attacks (red n=30, H-strain) and their subsequent relapses. **The relapses peak in May and in June, according to the timing of the majority of the primary attacks in September-October, but otherwise the curves are similar. Data compiled from Swellengrebel [17].

Among 30 patients of Korteweg [6,19], who were exposed naturally and came down with primary attacks in September and October (short latency!), 67 relapses were recorded after quinine treatment, from November of that year to December of the following year, with a peak in June. Swellengrebel pointed out that the two relapse distributions after heavy and low autumn infections had an almost identical shape and timing (Figure 1). Even though two different strains were compared and sporozoite inocula probably were different, he concluded that next year’s attacks of the natural Dutch strain would all be plain relapses. Implicitly it follows that a first attack after protracted incubation and its eventual relapses fell into the same pattern. 

Splitting the primary cases into early ones (summer, before August) and late ones (autumn, from September onwards, when mosquitoes appear in houses and become infected), the following pattern emerged. Of 242 naturally infected primary cases in summer (i.e. after long latency) 51% gave a relapse, and of those 48% more than once, whilst of 77 autumn primaries (after short latency) 49% relapsed and of those 71% more than once. In other words, the shorter the time between infection and primary attack, the higher the chances of repeated relapses. From this, Swellengrebel postulated that the inoculum size of sporozoites determines the number of relapses.

Earlier data of Korteweg correlated with this statement. He had found increasing numbers of relapses at the onset of a natural epidemic [6,22]. More cases relapsed in the following year during an emerging epidemic (supposedly with more sporozoites inoculated). And when the epidemic subsided, fewer cases relapsed the following year.

Moreover, Korteweg had found that relapsing malaria produced more gametocytes (75%) than primary cases (56%) and in higher densities [23]. The former group, because of the more benign symptoms, was less likely to see a doctor and gametocytes continued to circulate. Korteweg used his analysis of primary cases and relapses from the previous year to predict the incidence of the next year [4]. Though not explicitly stated, these observations fit with the idea that numbers of sporozoites determine the course of infections.

There was also an indication found in human behaviour. Naturally exposed patients in mental institutions in malaria regions showed a similar pattern [24]. The infection frequency in such institutions (in Medemblik, North-Holland, and Franeker, Friesland, and despite screened windows) was remarkably higher than among personnel and people in the towns, and therefore suitable for study. The relapse rate after quinine treatment of 84 patients was 69% and the primary attacks after September all relapsed in the spring or summer of next year. The researchers explained the higher relapse rate among mentally disturbed patients tentatively by reduced activity, as compared with alert healthy people. This allowed more mosquitoes to bite and to inject more sporozoites.

In conclusion, the number of biting infective mosquitoes (and thus, the inoculum size of sporozoites) determines the timing of primary attacks as well as the frequency of relapses, which in turn determines the outcome of next year’s incidence of clinical malaria.

Not only the relapse rate but apparently also the number of relapses per person was directly related to the transmission event. A first relapse after treatment of a short latency attack appeared after a period approximately similar to the duration of the long pre-patent period, which suggests the influence of internal or external factors.

I would like to point out the fact that the data of the Dutch investigators were scattered, and that, though on the right track, they did not thoroughly investigate the biological phenomena of inoculum size, latency and relapses within the possibilities of their time.

With reference to the mosquito-transmitted Dutch isolates, and the one from Madagascar, the term “strain” was less strictly described than a modern definition would require. However, the parasites, maintained through many GPI-patients and reared mosquitoes, may have been genetically more homogeneous than those transmitted “in the wild”. Infection of volunteers and patients in the past was less restricted by uniformity, regulations and ethical clearance or informed consent, despite a case fatality of eight per cent (62/807) among GPI-patients infected with the M-strain and left untreated under good medical care. Apparently, *P. vivax* infections are less innocent than is often assumed, at least in patients who suffer from an underlying illness, such as those with GPI [25].

To conclude this somewhat erratic collection of dispersed data and anecdotes from Dutch sources, a few items need to be discussed and expanded.

## Discussion

In 1946, Shute in England confirmed the Dutch findings on sporozoite inocula and incubation time. Pursuing this line of work started by the then deceased Dr. James with GPI-patients, he suggested that fewer than 2000 dissected and injected sporozoites (of a Romanian *P. vivax* strain with long latency) failed to give a short incubation [26]. However, it was not until 1976 that Shute, Garnham and colleagues performed the decisive experiments by injecting graded numbers of sporozoites of a North Korean long latency strain intradermally into mental hospital patients in Romania [27]. They confirmed the idea that low numbers of this temperate zone *P. vivax* led to long latency (1000 sporozoites and less), whilst the tropical Chesson strain always resulted in short latency, irrespective of the numbers of sporozoites injected. The authors calculated that the Korean strain used in this experiment produced one in a thousand sporozoites that gave a clinical attack with short latency and 999/1000 induced long latency. However, they did not consider the difference in (genetic) background of the few oocysts per mosquito (see my calculation above).

Short and Garnham, who revealed the existence of hypnozoites, assumed that *P. vivax* sporozoites of the temperate zones are programmed differently. Mosquitoes may have picked up a clone of parasites programmed either for long or for short latency. If we consider the existence of clones in nature unlikely, at least a minority of the oocysts growing from zygotes has the trait of short latency and thus, with more mosquitoes biting, and more short-type sporozoites in the inoculum, the chance of developing a parasitaemia at short notice increases. The environment of the parasite in local *An. maculipennis atroparvus* (the definitive host) influencing the genetic recombination, leading to early or late primary attacks and their eventual relapses, may also be considered.

Tiburskaja *et al*. also concluded that the relapse rate of various long latency strains of *P. vivax,* used in psycho-neurological hospitals in Moscow, depended on the number of mosquitoes used [28]. Patients with a short latency primary attack after exposure to enough biting mosquitoes are likely to relapse again after long latency. In this case the H-strain has similarities with the St. Elizabeth strain from temperate climates in North America, suggesting a complex genetic make-up of the non-tropical *P. vivax*[17,29]. In an attempt to explain the remarkable autumn peaks of Dutch “febris tertiana” of the 19^th^ century, which were virtually unknown in the 20^th^ century, Swellengrebel even considered the possibility that the Dutch strain was flexible, and would have produced mainly short incubations during that era [9,17].

We now know that sporozoites of *P. vivax* reside in parenchymal liver cells and multiply directly, or go into dormancy. Hypnozoites reactivate and multiply at later points in time. The phenomenon of primary attacks and relapses can be explained by the assumption that each one is a primary outbreak in itself, originating from genotypically different hypnozoites after varying periods of dormancy.

Dutch primary cases in autumn had more chance of relapsing than primary cases in spring or summer. The latter relapsed generally in the same year, but relapses from autumn primaries delayed until the next year. Thus, the period of December to April was virtually devoid of parasitaemias and clinical attacks. The reactivation of dormant liver stages seems to occur after a fixed time interval, and along the same line, the long latency of about 38–40 weeks requires some signal, either from the host or from beyond. The trigger for reactivation of hypnozoites is still enigmatic, but White [9] suggested convincingly that after a first clinical attack in long latency infections, subsequent relapses are induced by the preceding illness; he based this on the predictable periodicity (see Swellengrebel’s own pattern of relapses).

Factors responsible for activation of dormant liver forms by the human environment (the intermediate host) are also a possibility, but the Dutch malariologists had very restricted means to investigate this. For example, there are casuistic indications that stress, shock, physical weakness or confinement can provoke a relapse of tertian malaria (it was for good reason that Swellengrebel was concerned about his pregnant wife!). In The Netherlands, the story goes that a boy, cracking though the ice of a recently frozen canal, developed malaria. Major trauma or surgery of people from areas endemic for (vivax) malaria cause hormonal and cytokine changes which likewise may induce a relapse. Disturbance of the (hormonal) equilibrium may reactivate dormant hypnozoites “before their time”, which would imply the environment as the determining factor for the length of the period in the liver. However, later experimental attempts to provoke a clinical attack in Dutch GPI-patients failed [30].

Next to the likelihood of different genetic constitutions of sporozoites (and hypnozoites), it is also tempting to assume that the hormonal equilibrium of the human body causes liver-stage parasites to remain quiescent during autumn and winter. And when the body adjusts to more daylight, the dormancy ends, relapses and long latency primaries constitute the peak of primary clinical malaria (in The Netherlands in late spring). The observation that the relapse rate in naturally acquired infections was higher than with GPI-patients infected throughout the seasons [13] supports this possibility.

A new idea is that of a supposed influence of the many bites of the newly-emerged anopheline generation in the summer season. Their saliva might induce a reactivation of dormant liver forms that gives rise to relapses (with or without a primary attack within weeks of exposure in the previous year). In a recent interesting analysis of old time vivax malaria in Finland, Huldén *et al*. [31] applied the idea of Paul *et al*. [32] on the influence of mosquito saliva modulating malaria parasites in the human body. However, the amounts of mosquito bites in Dutch spring and early summer were almost negligible.

Modern methods of genotyping by analysing blood forms harvested at the primary attack, and those of relapses in the same patients, have shown that sporozoites in one inoculum, transferred by mosquito bite have made it likely that sporozoites have different genotypes [33,34]. Consequently, hypnozoites that give rise to relapses have a different make-up from liver forms that immediately proceed to schizonts, and thus, sporozoites may be of mixed genotype. If clonal hypnozoites become activated at predetermined intervals [35], they would not need an external trigger for activation.

## Conclusions

Observations made by Dutch malariologists in the past, suggested some sort of predetermination or clonation of *P. vivax* strains. Genetic differentiation must have established in zygotes and is expressed in oocysts, sporozoites and hypnozoites and determines the outcome, both in short or long latency and in relapse rates/numbers. In addition, factors of the host and the outside environment may determine the awakening of hypnozoites. A good understanding of experiments done in the past helps us to understand the epidemiology of present-day vivax-malaria, and assists in designing consequent interventions. Using these findings, one can come to conclusions based on careful analyses, as White did [9].

The observed difference in the clinical course among people with or without previous experience with the local *P. vivax* strain (intermittent or remittent/ tertian or quotidian fevers of Korteweg), and the well-known difficulty for microscopic diagnosis in the latter cases deserves serious exploitation in epidemiological studies or local diagnostic centres.

At present *P. vivax,* in all its geographical and genetic diversity, mainly allows us only to hypothesise about its biology. Apart from studying the enigmatic determinants that reactivate hypnozoites, a detection technique for the presence of one or more hypnozoites would be useful to make the blind use of a 14-day primaquine course obsolete. Further study is required of transmission, relapse patterns and timing in *P. vivax* strains from tropical and temperate zones.

## Competing interests

The author declares that he has no competing interests.
